# Consequences of a Human *TRPA1* Genetic Variant on the Perception of Nociceptive and Olfactory Stimuli

**DOI:** 10.1371/journal.pone.0095592

**Published:** 2014-04-21

**Authors:** Michael Schütz, Bruno G. Oertel, Dirk Heimann, Alexandra Doehring, Carmen Walter, Violeta Dimova, Gerd Geisslinger, Jörn Lötsch

**Affiliations:** 1 Institute of Clinical Pharmacology, Goethe - University, Frankfurt am Main, Germany; 2 Fraunhofer Institute of Molecular Biology and Applied Ecology - Project Group Translational Medcine and Pharmacology (IME-TMP), Frankfurt am Main, Germany; Duke University, United States of America

## Abstract

**Background:**

TRPA1 ion channels are involved in nociception and are also excited by pungent odorous substances. Based on reported associations of *TRPA1* genetics with increased sensitivity to thermal pain stimuli, we therefore hypothesized that this association also exists for increased olfactory sensitivity.

**Methods:**

Olfactory function and nociception was compared between carriers (n = 38) and non-carriers (n = 43) of *TRPA1* variant rs11988795 G>A, a variant known to enhance cold pain perception. Olfactory function was quantified by assessing the odor threshold, odor discrimination and odor identification, and by applying 200-ms pulses of H_2_S intranasal. Nociception was assessed by measuring pain thresholds to experimental nociceptive stimuli (blunt pressure, electrical stimuli, cold and heat stimuli, and 200-ms intranasal pulses of CO_2_).

**Results:**

Among the 11 subjects with moderate hyposmia, carriers of the minor A allele (n = 2) were underrepresented (34 carriers among the 70 normosmic subjects; p = 0.049). Moreover, carriers of the A allele discriminated odors significantly better than non-carriers (13.1±1.5 versus 12.3±1.6 correct discriminations) and indicated a higher intensity of the H_2_S stimuli (29.2±13.2 versus 21±12.8 mm VAS, p = 0.006), which, however, could not be excluded to have involved a trigeminal component during stimulation. Finally, the increased sensitivity to thermal pain could be reproduced.

**Conclusions:**

The findings are in line with a previous association of a human TRPA1 variant with nociceptive parameters and extend the association to the perception of odorants. However, this addresses mainly those stimulants that involve a trigeminal component whereas a pure olfactory effect may remain disputable. Nevertheless, findings suggest that future TRPA1 modulating drugs may modify the perception of odorants.

## Introduction

Genetic mutations in ion channels encompass a diverse range of pathological conditions including extreme pain conditions such as total insensitivity to pain or exaggerated paroxysmal pain [1]. A rare gain-of-function mutation in TRPA1 transient receptor potential cation channels causes the familial episodic pain syndrome [2]. TRPA1 is expressed on nociceptive neurons [3], [4]. They are gated by noxious cold (<17°C [4]), although this is species-specific [5] and can be switched to heat sensitivity by changes in N-terminal ankyrin repeat-rich domain [6].

Due to their role in pain perception, TRPA1 channels are reckoned as a promising novel target for new analgesics [7]. However, the function of TRPA1 may exceed the somatosensory system. In fact TRPA1 channels are expressed in the olfactory epithelium [8] and the olfactory bulb [9] of mice. This raises the possibility of a specific involvement of TRPA1 in olfaction. In addition, among activators of TRPA1 channels are also pungent odorous chemicals [7], [10]. This further suggests a role of TRPA1 in perception of odors with a trigeminal component. This is also supported by *in vitro* observations of TRPA1 activation by odors [11].

To address an involvement of TRPA1 in human olfaction, the present study employed a genetic approach based on the hypothesis that positive associations should then be multisensory. Specifically, gain-of-function *TRPA1* variants that increase the sensitivity to pain stimuli should also increase the sensitivity to odorous stimuli. A suitable variant accommodating this approach was found with the *TRPA1* rs11988795 G>A single nucleotide polymorphism. It has been associated previously with an increased sensitivity to nociceptive stimuli [12], however, without molecular proof yet. The present work pursued the hypothesis that this enhanced sensitivity will extend to olfactory stimuli.

## Methods

### Subjects and Design

The study followed the Declaration of Helsinki and was approved by the Ethics Committee of the Goethe-University Frankfurt am Main, Germany. Informed written consent from each participating subject had been obtained. The study largely paralleled a similar assessment of a linkage between olfaction and nociception via a Na_v_1.7 sodium channel genotype [13], without overlapping subjects. It was double blind with respect to the subjects’ *TRPA1* genotype. The subjects’ actual health was assessed by medical history, physical examination including vital signs, and routine clinical laboratory test results. Exclusion criteria were a current clinical condition affecting pain or olfaction, any other actual diseases and drug intake within a week except oral anticonceptionals.

Power calculations based on published data [14], [15] had resulted in a sample size of 80. Due to the enrolment of three non-Caucasian subjects and one additional Caucasian, 84 subjects were enrolled, which was covered by the Ethics approval. The cohort submitted to analysis consisted of a random sample of 38 unrelated healthy men and 43 unrelated healthy women (age 20–30 years, mean ± standard deviation 24.1±2 years, 13 smokers) of Caucasian ethnicity by self-assignment. The minor rs11988795 A allele was carried by 36 subjects (allelic frequency 26.5%), was equally distributed among genders (cross-tabulation: likelihood ratio = 0.344, p = 0.38) and the distribution of 45 non-carriers, 29 heterozygous and 7 homozygous carriers corresponded to the Hardy-Weinberg equilibrium (χ^2^ goodness of fit test: p = 0.54). The tests were done in the succession pressure pain, heat pain, cold pain, application of menthol, olfactory testing (Sniffn’ sticks), cold menthol pain, electrical pain, and intensity ratings of CO_2_ and H_2_S intranasal stimuli (for method details, see below).

### Genotyping

Genomic DNA was extracted from 200 µl blood using the EZ1 DNA Blood 200 µl Kit on a BioRobot EZ1 Workstation (Qiagen, Hilden, Germany). Genotyping for *TRPA1* rs11988795 G>A was done after the end of the study from genomic DNA by means of Pyrosequencing assays on a PSQ 96 MA System (Qiagen, Hilden, Germany) using the PyroMark Gold Q96 Reagents set (Qiagen, Hilden, Germany). PCR reactions were performed in a 25 µl assay volume on a Mastercycler ep gradient S instrument (Eppendorf, Hamburg, Germany), using the HotStar plus Taq Polymerase system (Qiagen, Hilden, Germany) and SNP-specific PCR primers (forward primer: 5′-TAAGTGAGCCAAGTTCAGATCAGA-3′ and reverse primer: 5′-biotin-TTTCACAGAAAGTGAGGTGTTGTA-3′). The PCR was done with an initial denaturation step for 5 min at 95°C, 50 cycles with a 30 second denaturation step at 95°C, an annealing step at 45°C for 30 seconds and an elongation step at 72°C for 30 seconds, followed by a final elongation step at 72°C for 5 min. The PCR product (25 µl) was used in the Pyrosequencing analysis as described previously [16] with the sequencing primer 5′-TGATCCTTCTTTTCTCAGTA-3′. Three samples of each genotype were also sequenced by an external provider (LGC GmbH, Berlin, Germany), using the conventional capillary sequencing method [17] on a ABI 3730 XL device (Applied Biosystems, Darmstadt, Germany), and implemented as positive controls during Pyrosequencing.

### Assessment of Olfactory and Nociceptive Function

#### Olfactory testing

The olfactory test was based on felt-tip pens that contained a solution of an odorant instead of liquid dye (“Sniffin’ Sticks”: Burghart, Wedel, Germany [18]). The pen’s cap was removed by the experimenter for approximately 3 s and the pen’s tip was placed 1–2 cm in front of the nostrils, in the case of triplet pen presentation at an interval of approximately 3 s. Three main components of olfactory function were assessed birhinally, namely the perception of odors at low concentrations, which is the odor threshold, the ability to recall an odor and name it, which is odor identification, and the distinction of different smells, which is the ability of odor discrimination.

#### Odor thresholds

Were obtained for the rose-like odor phenylethylethanol, which was presented in 16 successive 1∶2 dilution steps starting from a 4% solution (i.e., higher dilution steps mean less concentrated PEA). Using a three-alternative forced-choice task (3-AFC) and a staircase paradigm starting at low phenylethylethanol concentrations, one pen with the odorant and two blanks were presented at each dilution step. Two successive correct or one incorrect identification triggered the reversal of the staircase. The odor threshold was the mean of the last four out of seven staircase reversals.

#### Odor discrimination

Was determined with 16 triplets of pens, two containing the same odorant and the third a different, “target” one (i.e., (target/non-target) butanol/2-phenyl ethanol, isoamylacetate/anethole, anethole/eugenol, limonene/fenchone, (−)carvone/(+)carvone, eugenol/cinnamon aldehyde, dihydrorosenoxide/menthol, acetaldehyde/isoamylacetate, citronellal/linalool, pridine/limonene, limonene/citronellal, eucalyptol/dipyridyl, dipyridyl/cyclopentadecanoate, butanol/fenchone, octylacetate/cinnamon aldehyde, carvone/acetaldehyde). Using a 3-AFC paradigm, the subject’s task was to identify the target stick.

#### Odor identification

Was determined with 16 odors (i.e., orange, leather, cinnamon, peppermint, banana, lemon, liquorice, turpentine, garlic, coffee, apple, clove, pineapple, rose, anise and fish) using a 4-AFC task with presentation of a list of four descriptors for each pen. From the three olfactory subtests, normosmia, i.e., normal olfactory function, was established by calculating a composite “TDI score” (“**T**hreshold **D**iscrimination **I**dentification”) as the sum of the scores from the three subtests [19]. Normosmia is observed at TDI scores >30.5 whereas pathologic olfactory function is indicated by TDI ≤30.5, with the separation of hyposmia (30.5≥TDI>15.5) from functional anosmia at TDI ≤15.5 [14].

In addition to the olfactory test, suprathreshold olfactory stimuli were delivered to the nasal mucosa by means of an olfactometer (OM/2, Burghart Messtechnik GmbH, Wedel, Germany). Specifically, a Teflon tube (outer diameter 0.5 cm) was introduced approximately 1 cm into the right nostril. Through this tube, **pulses of H_2_S** (n = 18 stimuli, length 200 ms, concentration 5 ppm) were delivered directly to the mucosa [20] at 36–44 s intervals by embedding them in a constantly flowing airstream (8 l/min) at controlled temperature (36.5°C) and humidity (80% relative humidity). During this experiment the subjects were comfortably seated in an air-conditioned and visually shielded room; acoustic shielding was achieved with white noise (50 dB SPL) delivered via headphones and subjects observed a special breathing technique that avoids airflow in the nasal cavity (velopharyngeal closure [21]) to ensure correct stimulus delivery. The subjects performed a tracking task on a computer screen [22] where within 2.5 s after each stimulus a visual analog scale (VAS) was displayed to query ratings of the stimulus’ smell intensity (“no smell” to “maximum smell”). The median of the estimates of 18 single stimuli was submitted to statistical analysis.

#### Experimental pain testing

Pain thresholds to **blunt pressure** were obtained using a pressure algometer with a circular and flat probe of 1 cm diameter (Commander Algometer, JTECH Medical, Midvale, Utah). It was placed perpendicularly onto the mid-phalanx of the right middle finger. The pressure was increased at a rate of approximately 9 N/cm^2^ per second until the subject indicated pain. The increase in pressure was controlled manually by the investigator and stopped once the subject reported pain. The procedure was repeated five times at intervals of 30 s and the pain threshold was the median of the five measurements.

Pain thresholds to **electrical stimuli** were obtained using a constant current device (Neurometer® CPT, Neurotron Inc., Baltimore, MD) that delivered sine-wave stimuli at 5 Hz applied via two gold electrodes placed on the medial and lateral side of the mid-phalanx (middle finger of the right hand as default-testing site). Their intensity was increased from 0 to 20 mA by 0.2 mA/s. During the test, subjects kept a button continuously pressed until they felt pain and interrupted the current by releasing the button. Measurements were repeated five times at intervals of 30 s and the median of the electrical currents at which subjects had released the button was the pain threshold.

Pain thresholds to **cold stimuli** were obtained using a Thermal Sensory Analyzer (Medoc Advanced Medical Systems Ltd., Ramat Yishai, Israel). Cold stimuli were applied with the 3×3 cm^2^ thermode placed on a skin area of the left or right (randomized) volar forearm. The temperature was lowered from 32°C to 0°C by 1°C/s. The subject pressed a button at the first sensation of pain. Measurements were repeated five times and the median of these measurements was the threshold. Subsequently, a plaster soaked with menthol (2 ml of a 40% menthol dissolved in 90% ethanol) was applied onto the skin area for 30 min and cold pain measurements were repeated (cold/menthol pain thresholds).

Pain thresholds to **heat stimuli** were obtained using the same device as for cold stimulation, placing the 3×3 cm^2^ thermode on the contralateral volar forearm (to forearm used for cold pain measurements). Temperature was continuously increased from 32–52.5°C by 0.3°C/s and the subject pressed a button at the first sensation of pain. As previous experiments had shown that three heat applications are needed to reach a plateau in the thresholds, heat applications were repeated eight times and the median of last five responses was used as heat pain threshold.

Finally, the perceived intensity of chemical pain was queried by using CO_2_ (60% v/v, n = 18 stimuli) instead of H_2_S as olfactometer stimulus applied to the right nostril. CO_2_ is converted into bicarbonate and protons by the enzyme carboanhydrase [23], it evokes a short stinging pain sensation [24] due to excitation of trigeminal nociceptors [25], [26]. In analogy to the H_2_S-stimuli, the CO_2_ pulses were rated for their painfulness using visual analogue scales (“no pain” to “maximum pain”). The median of the estimates of 18 single stimuli was submitted to statistical analysis.

### Statistics

Olfactory test results (threshold discrimination, identification and intensity estimates of H_2_S stimuli) were standardized by z-transformation and submitted to single analysis of variance for repeated measures (rm-ANOVA) using “odor task” as within-subject factor (4 levels) and “genotype” as between-subjects factor. In addition, olfactory test results were compared separately between genotype groups by means of t-tests, without z-transformation. To accommodate the complexity of pain phenotypes by clustering as proposed previously [27]–[29], the otherwise similar analyses of the pain data included the additional factor “pain cluster”, which was obtained by Ward hierarchical clustering with the squared Euclidian distance and subsequent description by classification and regression tree (CART) classifiers. Specifically, rm-ANOVA was calculated with z-transformed pain parameters (n = 6, cold and menthol pain thresholds multiplied with −1 to obtain similarly directed measures), using “pain measure” as within-subject factor (6 levels) and “genotype”, and “pain cluster” as between-subjects factors. To check for gender differences in the genotypic effects, “gender” was introduced in a further combined analysis, however, at an exploratory level as the study was not powered for gender differences. Moreover, while the original study reporting a function of the *TRPA1* rs11988795 G>A variant also reported interactions in the modulation of pain between gender and several variants in other genes [30], gender interaction was not reported for *TRPA1* variants and therefore not a major factor to be introduced in the present study design. Further statistical analyses included cross-tabulations, rank correlations (Spearman’s ρ) and effect size calculations using Cohen’s d (values of 0.2, 0.5 and 0.8 being indicative of a small, medium and large effect size, respectively [31]). A gene dose effect was not assessed for reasons of statistical power (n_AA carriers_ = 7). Statistics were done using SPSS (version 21 for Linux, SPSS Inc., Chicago, USA) or R (version 2.14.1 for Linux; http://CRAN.R-project.org/); the α-level was set at 0.05.

## Results

### Olfactory Test Results

**Figure 1:**
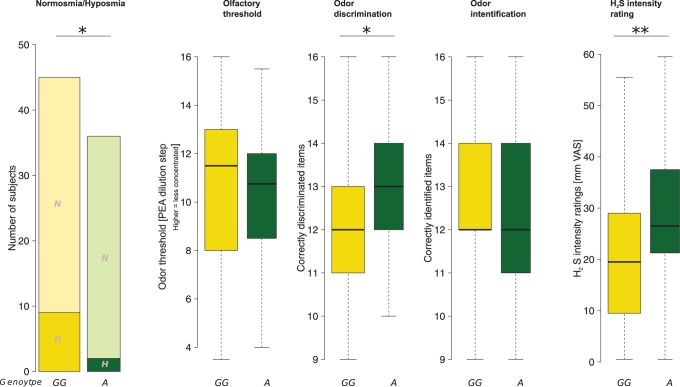
Olfactory parameters and their modulations by the *TRPA1* rs11988795 G>A genotype (wild type subjects, GG, versus carriers of the minor A allele). From left to right: (i) In cross-tabulation of normosmic (N) versus hyposmic (H) subjects, hyposmic subjects were underrepresented among the carriers of the A allele (likelihood ratio test: p = 0.049). (ii) This genotypic association was more produced in the performance in the odor discrimination subtest of the Sniffn’ Sticks test battery [18], where carriers of the A allele performed significantly better (t-test: p = 0.029). (iii and iv): The other olfactory tests, however, were unaffected by the *TRPA1* genotype (odor threshold and odor identification). (v) The genotype effects were most pronounced in the perceived intensity of olfactory H_2_S stimuli administered with an olfactometer (p = 0.006). The widths of the bars or boxes are proportional to the respective numbers of subjects per group. *: p<0.05, **: p<0.01. The minimum, quartiles, median (solid horizontal line within the box), and maximum are used to construct the box plots.

None of the subjects reported any perception of pathological pain or smell sensitivities. Most subjects (n = 70) were normosmic as indicated by a TDI (“**T**hreshold **D**iscrimination **I**dentification”) score ≥30.5 [14]. Neither the subjects’ sex (χ^2^ test: p = 0.45) nor their smoking habits were associated with the diagnosis of hyposmia (χ^2^ test: p = 0.92). However, the *TRPA1* rs11988795 A allele was rarer among the 11 hyposmic subjects (TDI of 24.75–30.75). Specifically, the minor allele was carried by two (18.2%) of the 11 hyposmic subjects and by 34 (48.6%) of the 70 normosmic subjects (cross-tabulation: likelihood ratio = 3.87, p = 0.049; Figure 1).

Odor task results were differently affected by the *TRPA1* genotype (rm-ANOVA effect “odor task”: df = 3,237, F = 0.041, p = 0.99, interaction “odor task” by “genotype”: df = 2,237, F = 3.34, p = 0.02; with a tendency toward significant between-subject effects of “genotype”, df = 1,79, F = 3.213, p = 0.077). Additionally introducing “gender” into the analysis failed to produce significant effects (p>0.05 for all main effects and interactions involving “gender”). In the post hoc tests, better olfactory performance in carriers of the *TRPA1* rs11988795 A allele was observed with odor discrimination (t-test: p = 0.029; 13.1±1.5 versus 12.3±1.6 correct discriminations, Cohen’s d = 0.5). Of note, of the 16 odor discrimination items, butanol (target) could be discriminated from fenchone (non-target) by 35 of the 36 A allele carriers but by only 34 of the 45 wild type subjects, which was significant after α correction (cross-tabulation: likelihood ratio = 8.76, p_ = _0.003). The most pronounced genotype effect was observed on the perceived intensity of the H_2_S stimuli. Specifically, carriers of the A allele perceived the H_2_S stimuli significantly more intense than non-carriers (29.2±13.2 versus 21±12.8 mm VAS, t-test: p = 0.006, Cohen’s d = 0.63), which persisted after α correction (p = 0.024). The significance of this effect also persisted when excluding hyposmic subjects from the analysis (p = 0.044). Moreover, the ratings of the H_2_S stimulus intensity correlated with the perceived cold (Spearman’s ρ = 0.28, p = 0.012, and ρ = 0.27).

### Nociceptive Test Results

**Figure 2:**
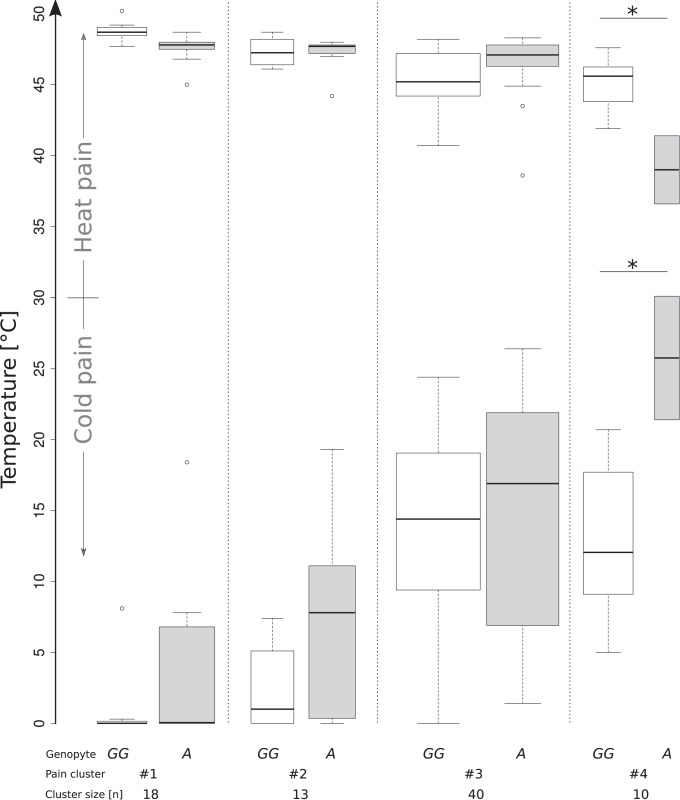
Experiment to re-establish the previously observed association of the *TRPA1* rs11988795 G>A variant with cold (and heat) pain [12]. A statistically significant genotype effect on heat and cold pain thresholds (ANOVA factor “genotype”) was most pronounced in cluster #4 characterized by a high heat pain sensitivity (Table 1), which was reflected in a significant ANOVA interaction “genotype” by “pain cluster” for heat pain thresholds. The minimum, quartiles, median (solid horizontal line within the box), and maximum are used to construct a “box and whisker” plot.

In contrast to olfaction, nociception was not modulated by the *TRPA1* genotype in the whole cohort (p = 0.385–0.757 for the various nociceptive measures, Cohen’s d = 0.07–0.196). Therefore, genetic modulations were searched in subgroups of subjects. Four pain sensitivity subgroups were identified (Table 1 and Supporting Figure 1). The *TRPA1* variant exerted distinct genotype effects on different pain parameters (rm-ANOVA effect “pain measure”: df = 5,365, F = 2.596, p = 0.025, interaction “pain measure” by “pain cluster”: df = 15,365, F = 14.12, p<0.001, effect “genotype”: df = 1,73, F = 10.138, p = 0.001, interaction “genotype” by “pain cluster”: df = 3,73, F = 3.205, p = 0.018; further, non-significant, effects and interactions not shown). In analyses of single pain stimuli, cold (ANOVA factor “genotype”: df = 1,73, F = 8.305, p = 0.005, Cohen’s d = 0.53, 0.9, 0.12 and 2.5 in clusters #1–#4, respectively) and heat (df = 1,73, F = 9.272, p = 0.003; Cohen’s = 1.4, 0.17, 0.48 and 3.2 in clusters #1–#4, respectively) pain thresholds were modulated by *TRPA1* rs11988795 G>A (Figure 2). The modulation of heat sensitivity was not uniform across clusters (interaction “genotype” by “pain cluster”: df = 3,73, F = 7.056, p = 0.0003). Other pain readouts including the intensity of CO_2_ were unaffected by the *TRPA1* genotype (48.2±16.8 mm VAS in A allele carriers versus 51.4±15.6 mm VAS in non-carriers, t-test: p = 0.39). Additionally introducing “gender” into this analysis failed to produce significant effects (p>0.05 for all main effects and interactions involving “gender”).

**Table 1:** Decision rules (separated by lines) extracted from the CART classifier, providing a semantic description of the pain phenotypes (clusters #1–#4) found by Ward cluster analysis.

Case belongs to		IF (Rule conditions)
Cluster #1:	(IF	CO_2_ intensity ≤42. 5 mm VAS
	AND)	Heat threshold >46.55°C
	OR (IF	42.5 mm VAS<CO_2_ intensity ≤50.25 mm VAS
	AND)	Pressure pain threshold >27.25 N/m^2^
Cluster #2:	IF	CO_2_ intensity >50.25 mm VAS
	AND	Pressure pain threshold >27.25 N/m^2^
Cluster #3:	IF	CO_2_ intensity >42. 5 mm VAS
	AND	Pressure pain threshold ≤27.25 N/m^2^
Cluster #4:	IF	CO_2_ intensity ≤42. 5 mm VAS
	AND	Heat threshold ≤46.55°C

The CART identifiers of the cluster membership correctly assigned 95.1% of the subjects.

## Discussion

The *TRPA1* variant rs11988795A was associated with enhanced perception of odorous stimulants. The direction of this genotypic effect toward enhanced sensitivity agrees with the expectation from a previous observation, namely that the same variant enhanced sensitivity toward nociceptive cold stimuli [12]. This suggests that a functional association of the human *TRPA1* variant is not restricted to the perception of pain but extends to odour perception. However, other than with Na_v_1.7 variantswhere effects of a gain-of-function variant in the sodium channel gene *SCN9A* [13] can be attributed to the olfactory system, with TRPA1 a trigeminal component of the observed genetic modulation of odorant perception needs clearly to be considered.

Several substances that evoke an olfactory sensation have been shown to excite TRPA1 channels. This particularly applies to butanol for which the genotype effect on its discrimination from fenchone was presently observed. Butanol is a primary alcohol which at 1 mM applied to HEK293 cells expressing hTRPA1 increased Ca^2+^ currents [32]. However, the present study was not sufficiently powered to observe patterns in the correct discriminations of 16 target/non-target odor combinations and therefore, statistical findings have to be regarded with caution. A presence of TRPA1 genotypic effects for TRPA1 excitants cannot be claimed from the present observations. Several further odorants have been associated with TRPA1 excitation such as eugenol [7] and amylacetate [11], however, this did not provide a clear pattern with respect to genotype differences in odor discrimination. In addition to the insufficiency of the power, the relative excitation by butanol and other excitants presently cannot be judged since the observations originate from different studies [11], [32]. As n-butanol had been used as test substance for olfactory thresholds in the first version of the Sniffn’ Sticks test (e.g. [14]) before it had been replaced with phenyl ethyl ethanol, a possibility that the observed effects were related via the olfactory rather than the trigeminal system remains. However, while this would receive support by the expression of TRPA1 in the olfactory epithelium and olfactory bulb in mice, being specific olfactory structures, the translation to humans remains uncertain. TRPA1 is absent from the human olfactory bulb [33], [34] and the expression pattern in the human olfactory epithelium is unknown. Therefore, concluding a purely olfactory effect in humans would stand on weak grounds. Moreover, the olfactory bulb is in close relation with trigeminal afferents [35]–[37] which further impedes a clear distinction of the sensory qualities affected by the *TRPA1* variant. Finally, olfactory tests results are known to be influenced by cognitive factors [38], [39], which had not been acquired presently. However, considering these factors as a potential confounder of the present results would imply that the *TRPA1* variant had an influence on them, for which no clear mechanistic hypothesis exists.

The perception of the smell of terpineol, amylacetate, benzaldehyde and toluene involves TRPA1 excitation, however, they seem to be detected as trigeminal irritants [11]. Therefore, the presently observed changes in olfactory measures might have been conferred via a trigeminal mechanism. This would explain the lack of *TRPA1′s* genetic association with odor thresholds, which had been obtained using the purely olfactory stimulant phenylethylethanol. Apparently contradicting this hypothesis is the observed increase in the perception of H_2_S being a pure olfactory stimulus that cannot be perceived by anosmic subjects [40], [41], thus providing that no unwanted trigeminal co-stimulation had taken place. To pursue the hypothesis of a trigeminal component, we performed an additional experiment. Specifically, seven subjects received 15 stimuli of clean air (200 ms, ISI 20–30 s) and 15 no-stimuli, i.e., no olfactometer action, at random order. The question, presented within 2–5 s after each stimulus, whether or not they had received a stimulus was correctly answered 30, 28, 24, 24, 26, 17 and 18 times by subject 1–7, respectively, which was always more often than by pure chance (15 correct responses; χ^2^ test: p<0.0001). This indicated trigeminal co-stimulation and further strengthens the association of the observed effects with the trigeminal rather than olfactory sensory system. However, it was very slight and it was only detected when specifically addressed, not during the main experiments. A slight trigeminal co-stimulation is known to play a key role in the unconscious detection of odors [42]. As this effect has been described to occur more at a subconscious level [42], its absence on suprathreshold trigeminal CO_2_ stimuli might be explicable. Thus, the unintended trigeminal co-stimulation provides an explanation of the increased H_2_S intensity. It is better compatible with the absent effect on odor threshold than a direct *TRPA1* effect on olfaction, which however remains an alternative possibility when assuming that H_2_S is an excitant of TRPA1, in contrast to phenylethylethanol. This is conceivable as odorants differ in their effects on TRPA1 channels [11]. However, a direct involvement of TRPA1 in human olfaction is conceivable although current knowledge provides no clear proof. That is, TRPA1 expression in the olfactory epithelium [8] and olfactory bulb [9] has been shown to occur in mice. In the human olfactory bulb, however, TRPA1 was neither found by a proteomic analysis [33] nor by mRNA quantification on a microarray [43]. Whether it is expressed in the human primary olfactory neurons remains to be shown.

The clustering analysis identified a subgroup of subjects sharing similar pain phenotypic patterns. On one of these patterns, the *TRPA1* variant could be associated with different sensitivity to thermal pain among carriers and non-carriers. However, the clustering resulted in a split of the sample into smaller subsamples which jeopardized the statistical power. On this basis, the present results regarding nociceptive signals have to be viewed with reservation although the genetic association with pain was implemented mainly to address compatibility with previous results [12]. It has to be noted that the reproduction of the mentioned results was not exact as the original association was observed in heterozygous subjects whereas in the present analysis, heterozygous and homozygous carriers of the variant *TRPA1* rs11988795 A were pooled to maintain statistical power. A nevertheless performed analysis with three genotype groups showed a significant between-subjects effect of “genotype” (df = 2,69, F = 8.26, p = 0.001). Moreover, contrasting heterozygous carriers with non-carriers while leaving out the homozygous carriers also showed a significant genotype effect (df = 1,66, F = 9.705, p = 0.003) stratified for pain clusters (interaction “genotype” by “pain cluster”: df = 3,66, F = 3.853, p = 0.013). However, in the whole cohort no strong association with pain readouts could be observed. As we have recently demonstrated [44], clustering pain phenotypes provides larger genetic effect sizes, which in the present analysis had to be obtained with another commonly recommended [45] clustering strategy due to the small sample size. Indeed, clustering strategies are increasingly pursued in pain research [29], [45] as they fulfill the assumption that different pain phenotypes are based on different molecular pathomechanisms that are accessible to specific treatments. Reproduction of non-clustered data have often failed (e.g., [46] versus [47]), not necessarily because of non-functionality of the genetic variants. In a randomly chosen sample, the pattern of functional variants that are concomitantly present with the variant of interest can be also as such that a cancelling-out of the effects occurs [48]. This leads to small overall effects sizes [49] while greater effects apply to subgroups [50]. A consequence of the need for clustering is that genetic association studies in pain require an increasing number of participants. The statistical power effect further decreases when gender becomes an additional factor modulating the genotype effect, which in a previous study, however, has been shown to not apply to the present *TRPA1* rs11988795 G>A variant [30] but in the same study associations of variants in other genes were modulated by the subject’s gender (e.g., variants in *TRPV1*, *OPRD1*, *COMT*).

Interestingly, the present finding reproduced the previous observation of an association of the *TRPA1* rs11988795 variant with different sensitivities to thermal pain. Specifically, carriers of the minor A allele had a shorter withdrawal latency to noxious cold and in a small subgroup, the allele was also associated with differences in the sensitivity to heat pain. Indeed, role of TRPA1 channels in thermal sensation is controversially discussed [51]. The observation of an association with heat pain had been previously interpreted on the basis of an earlier reported observation that local capsaicin administration affected the cold sensation longer than any other sensations including heat [52]. Those observations point at interactions among TRP channels [53]. TRPA1 channels are often co-expressed with heat (>43°C [54]) gated TRPV1 [4], [55]) and the channels act in concert [56]. That is, TRPV1 can oligermerize with other TRP family subunits including TRPV3 and TRPA1 [57]. The heteromerization between TRPV1 and TRPA1 channels can substantially affect the calcium signaling pathways of TRPA1 homomers [57] and the co-expression can lead to outward rectification of single channel current-voltage relationships and modulation of open probabilities [58]. Moreover, while thermal (heat) hyperalgesia was initially attributed solely to TRPV1, currently TRPA1 and TRPV1 are regarded to be interdependently regulated downstream of PLC-coupled bradykinin (BK_2_) receptors [59] to establish hypersensitivity to heat [56].

It has finally to be noted that the molecular mechanism of functional associations of *TRPA1* rs11988795 is still unknown. The SNP is located in intron 20–21 of the *TRPA1* gene (Ensembl transcript ID ENST00000262209) with a distance of 618/908 nucleotides from exon boundaries. In the HapMap [60] CEU cohort, it is located in a 25 kb haploblock spanning chromosome 8 position 73102904 (rs12550748) to 73128527 (rs3735942) (Supporting Figure 2). This haploblock contains two splice sites (rs3824151 and rs35427625) and one synonymous (rs13280644 G>A; L830L) variant. A *TRPA1* splice effect associated indirectly with rs11988795 may provide a hypothesis for the molecular background of the so far observed functional associations of this variant.

## Conclusions

Present results augment the body of evidence about the genetic modulation of the perception of odors in the average population. To the genetic modulation of odorant perception seems to contribute the modulation of a trigeminal component, however, with weaker support a direct interaction with an olfactory component seems also conceivable. Following the present positive evidence, future elaboration of *TRPA1* genetic effects on odor perception with a focus on TRPA1 activating odorants remains a future scientific task. From a pharmacological point of view, the results indicate that modulation of TRPA1 may, beside deactivation for the treatment of pain, also be directed toward activation to ameliorate diminished olfaction for which a medical need exists [61]. It has been shown that this goal may be achievable by enhancement of trigeminal excitatory input to the olfactory system [37] and therefore does not crucially require olfactory specificity.

## Supporting Information

Figure S1 **Heat plot and cluster dendrograms of the individual responses to pain stimuli.** More intense orange coloring indicates higher pain sensitivity equal to lower pain thresholds. Ward clustering suggested two or four pain sensitivity subgroups (Figure 2). As the latter singled out subjects with censored cold or cold/menthol thresholds (0°C) in a separate cluster, the four-cluster solution was preferred as it was not substantially worse that the two-cluster solution as assessed by means of Silhouette plots (not shown).
(EPS)

Figure S2 **Haploblock structure and SNP localization of the** ***TRPA1*** **gene of the HapMap CEU population (Utah residents with Northern and Western European ancestry;** http://hapmap.ncbi.nlm.nih.gov/ **).** The *TRPA1* rs11988795 G>A SNP (framed red) is located in a haploblock that comprises many more SNPs. While rs11988795 is intronic but not in a splice site, the haploblock contains 2 splice site SNPs (rs3824151 and rs35427625) that according to this analysis are the most likely candidates to confer the repeatedly observed functional association of the SNP. Top: SPNs of the dbSNP database in the region of the identified *TRPA1* haploblock that contains rs11988795. Bottom: Haploblock structure according to a HaploView analysis, using the 95% confidence intervals on D’ [62] method as the default of the HaploView software [63] and showing the linkage between SNPs, those in the HapMap data set are given at the top of the haploblock structure.
(EPS)
